# One-Step Synthesis of Microporous Carbon Monoliths Derived from Biomass with High Nitrogen Doping Content for Highly Selective CO_2_ Capture

**DOI:** 10.1038/srep30049

**Published:** 2016-08-04

**Authors:** Zhen Geng, Qiangfeng Xiao, Hong Lv, Bing Li, Haobin Wu, Yunfeng Lu, Cunman Zhang

**Affiliations:** 1 Clean Energy Automotive Engineering Center, Tongji University, Shanghai 201804, China; 2 School of Materials Science and Technology, Tongji University, Shanghai 201804, China; 3 Research & Development Center, General Motors, MI 48265-3300, United States; 4 School of Automotive Studies, Tongji University, Shanghai 201804, China; 5 Department of Chemical and Biomolecular Engineering, University of California, Los Angeles, CA 90024, United States

## Abstract

The one-step synthesis method of nitrogen doped microporous carbon monoliths derived from biomass with high-efficiency is developed using a novel ammonia (NH_3_)-assisted activation process, where NH_3_ serves as both activating agent and nitrogen source. Both pore forming and nitrogen doping simultaneously proceed during the process, obviously superior to conventional chemical activation. The as-prepared nitrogen-doped active carbons exhibit rich micropores with high surface area and high nitrogen content. Synergetic effects of its high surface area, microporous structure and high nitrogen content, especially rich nitrogen-containing groups for effective CO_2_ capture (i.e., phenyl amine and pyridine-nitrogen) lead to superior CO_2_/N_2_ selectivity up to 82, which is the highest among known nanoporous carbons. In addition, the resulting nitrogen-doped active carbons can be easily regenerated under mild conditions. Considering the outstanding CO_2_ capture performance, low production cost, simple synthesis procedure and easy scalability, the resulting nitrogen-doped microporous carbon monoliths are promising candidates for selective capture of CO_2_ in industrial applications.

Carbon capture and sequestration are effective solution for reducing anthropogenic CO_2_ emission^1^,^2^. Since combustion streams, such as flue gas emitted from coal-fired power plants, may comprise ~70% of N_2_ and 3–15% of CO_2_, selective capture of CO_2_ is highly desired^3^. Compared with the conventional amine-scrubbing and pressure-swing adsorption technologies^1^,^2^, selective physisorption techniques are more effective and environmentally friendly. To date, various porous solids, including porous carbons^4^,^5^,^6^,^7^,^8^,^9^,^10^,^11^, metal-organic frameworks (MOFs)^12^,^13^,^14^ and covalent organic frameworks (COFs)^15^,^16^ have been extensively studied. Numerous efforts have been focused on optimizing the surface area and pore structure, as well as enhancing their affinity to CO_2_ by incorporating various functional groups^4^,^5^,^6^,^7^,^8^,^9^,^10^,^11^,^12^,^13^,^14^,^15^,^16^. Various materials with selective CO_2_ capture capability have been prepared in laboratory scale; large-scale, low-cost and facile synthesis of materials for effective CO_2_ capture, however, remains challenging.

Chemical activation of biomass has been broadly adopted for large-scale and low-cost production of porous carbon materials for a wide range of applications^17^,^18^,^19^. However, such biomass-based carbons generally do not possess sufficient capability for selective adsorption of CO_2_^19^. It has been found that incorporating nitrogen (N)-containing groups into carbons (e.g., phenyl amine (Ph-NH_2_) and pyridine-N groups) can effectively improve their selective CO_2_ capture over N_2_ or CH_4_, mainly due to the preferred interactions between CO_2_ and the electronegative N-containing groups^4^,^5^,^6^,^7^,^8^,^9^,^10^,^11^. Generally, the doping is achieved by direct activation of N-containing biomass^20^,^21^,^22^,^23^ or by treating carbons with ammonia^24^,^25^. For the former approach, the N-containing moieties, however, are generally volatized during the activation process, resulting in carbons with low N content (e.g., <5 wt%). Similarly, the post-treatment process generally leads to carbons with low N content (e.g., <3 wt%) due to low reaction efficiency between the ammonia and the carbon scaffolds.

We report herein a novel synthesis of N-doped microporous carbon monoliths derived from biomass (corncob) using an ammonia gas (NH_3_)-assisted activation process, where NH_3_ serves as both the activating agent and the N source. As shown in Fig. 1, corncob particles were efficiently transformed to N-doped microporous carbon monoliths by one-step NH_3_-assisted activation process. Both pore forming and nitrogen doping simultaneously proceed during the process, obviously superior to conventional chemical activation. The dual role of NH_3_ as the activating agent and N source leads to high surface area, superior pore texture and high N content of the as-prepared carbon materials. To the best of our knowledge, such an NH_3_-assisted activation process with high-efficiency has not been reported yet. The resulting N-doped microporous carbon monoliths exhibit excellent selective CO_2_ capture performance with excellent CO_2_ selectivity over N_2_ of 82, which is the highest among reported nanoporous carbons.

## Materials and Methods

### Sample preparation

Nitrogen-doped active carbons were prepared by a novel chemical activation method using biomass corncob as the carbon source and NH_3_ as the activating agent and nitrogen source. Detailed procedures are described as follows. Firstly, after drying for 12 h at 120 °C, corncobs were grounded and sieved into powders with typical size of less than 880 μm. Secondly, the corncob powders were transferred to ceramic boats and heated to 400 °C at 5 °C min^−1^ under N_2_ flow of 1.5 L min^−1^ in a horizontal tube furnace to obtain the carbonized particles. Then N_2_ was switched to NH_3_ and the sample was continued to be heated at 400–800 °C under NH_3_ flow of 1.5 L min^−1^. Then NH_3_ was switched back to N_2_ when activation was completed and temperature was reduced to 400 °C. Finally, sample was obtained by lowering the temperature to room temperature under N_2_ atmosphere. The resulting N-doped active carbons are denoted as NAC-*x*-*y*, where *x* is the activation temperature (°C) and *y* is the activation time (hours) used, respectively.

Corncob-derived activated carbons (CACs) were prepared by KOH chemical activation with biomass corncob as carbon sources and KOH as the activating agent. Detailed procedures are described according to previous reports^17^.

### Materials characterization and analysis methods

The textural properties of the samples were performed by N_2_ sorption at 77 K using a Micromeritics ASAP2020 over a wide relative pressure ranging from about 10^−6^ to 1.0. Prior to the measurements, all samples were degassed at 300 °C for 10 h. The specific surface area (SSA) was assessed by standard BET method (software available in the ASAP2020) using adsorption data in the relative pressure ranging from 0.02 to 0.25. The total pore volume (V_t_) was calculated by converting the amount of N_2_ adsorbed at a relative pressure of 0.98 to the volume of liquid adsorbate. The micropore volume was calculated by the Dubinine Radushkevich (DR) equation. Pore size distributions (PSDs) were calculated by using the Density Functional Theory (DFT) Plus Software (provided by Micromeritics Instrument Corporation), which is based on calculated adsorption isotherms for pores of different sizes. In the DFT calculations, the equilibrium model of carbon slit-shaped pores with N_2_ sorption was applied.

Scanning electron microscope (SEM) and Energy Dispersive Spectrometer (EDS) mapping images were performed on a FEI NOVA Nano electron microscope. Transmission electron microscopy (TEM) and selected area electron diffraction (SAED) were carried out using JEOL JEM-2100F electron microscope. Elemental analysis was obtained by a Thermo Flash EA2000 elemental analyzer. Fourier transform infrared (FT-IR) spectroscopy for samples was analyzed through a BRUKER EQUI NO -XSS spectrometer using the attenuated total reflectance method. X-ray photoelectron spectroscopy (XPS) analysis was performed with an ESCALAB 250Xi spectrometer (Thermo Electron) using a monochromic Al Ka source at 1486.6 eV.

CO_2_ and N_2_ adsorption performance at ambient pressure was carried out on ASAP2020 adsorption analyzer. All of the samples were evacuated to ultrahigh vacuum at 300 °C for 10 h before the test. CO_2_ and N_2_ adsorption measurements were performed over the pressure range of 0–1.0 bar at 273 and 298 K, respectively. High purity CO_2_ and N_2_ (99.999%) were used throughout the tests.

Calculation of CO_2_/N_2_ selectivity: We calculated the initial slopes of gas uptake for both CO_2_ and N_2_. The ratio of the slopes was used for calculating the selectivity at 298 K.

Heat of CO_2_ adsorption calculation: The isosteric heat of adsorption (Q_st_) can be calculated using CO_2_ adsorption isotherms measured at different temperatures based on the Clausius-Clapeyron equation (Eq. 1):
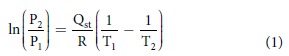
where P_i_ is pressure for isotherm i; T_i_ is temperature for isotherm i; R is the universal gas constant 8.315 J K^−1^ mol^−1^. In this study, the Q_st_ was calculated using CO_2_ adsorption isotherms measured at 273 and 298 K.

## Results and Discussion

**Figure 2:**
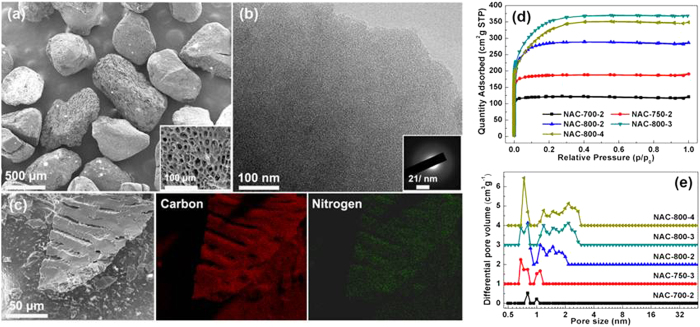
(**a**) SEM images, (**b**) TEM (inset: selected area electron diffraction pattern) and (**c**) EDS mapping images of typical sample NAC-800-3; (**d**) N_2_ sorption isotherms and (**e**) Pore size distributions of all NAC samples.

Figure 2 shows scanning electron microscope (SEM) and transmission electron microscopy (TEM) images of a typical sample (NAC-800-3). It presents the monolith shape with an average size of 0.5–1 mm in diameter (Fig. 2a), which would be more advantageous over smaller-size sorbents for practical applications^4^. The skeleton of these monoliths exhibits a honeycomb-like structure with interconnected macropores of ~15 μm in diameter (inset of Fig. 2a). Such pore structure is also advantageous for CO_2_ sorption with low transport resistance^5^. TEM image (Fig. 2b) suggests that the carbon contains uniform micropores. The electron diffraction of the sample shows an amorphous structure (inset of Fig. 2b), which is consistent with most of the activated carbons derived from biomass.

Figure 2c further shows a SEM image of a NAC sample along with its element mapping. Noticeably, N is homogenously distributed within the carbon framework. Elemental analysis (Table 1) shows negligible amount of N in natural corncob and its directly carbonized product. Meanwhile, activation in NH_3_ can easily dope N into carbon framework even at 400 °C, although it is difficult to form a porous structure at such a low temperature. Further increase of the activation temperature leads to increase of N content, reaching ~12 wt% at 800 °C, which is unsurpassable for all existing chemical activation methods (e.g., 5.11 at% for HHC^21^; 4.84 wt% for PA-400-KOH-1-600^23^; 8.1 wt% for CN700^24^; 9.2 wt% for CNO300^26^; 4.5 wt% for AN^27^).

Such high N content is comparable with those made from specially designed compounds that require highly complex chemical synthesis (e.g., 10.14 wt% for CP-2-600^5^; 12.9 wt% for IBN9-NCI-A^8^; 11.95 wt% for PPN-6-CH_2_DETA^14^). The increasing trend of N doping with increasing temperature is different from literatures, where the content of N doping generally decreases with increasing temperature, particularly, when temperature is above 500 °C due to high volatility of the N-containing species^6^,^28^. The continuous increase of the N content implies a dynamic balance of N doping and removal during the reactions, where the rate of N doping into carbon is over that of N removal from carbon. This method provides a direct approach to fabricate highly N-doped carbons simply by activating biomass in NH_3_ atmosphere.

Figure 2d shows N2 sorption isotherms of the samples prepared at different activation temperature and time, which exhibit type I isotherms with typical microporous structure^20^. With increasing the activation temperature from 400 to 800 °C, isotherms with significantly increased pore volume accompanied with enlarged pore sizes are obtained. As shown in Table 1, NAC-400-2, NAC-500-2, and NAC-600-2 show insignificant value of specific surface area (SSA), implying a poor activation at temperature below 600 °C. SSA gradually increases with increasing activation temperature and time, reaching a maximum of 1154 m^2^ g^−1^ with an activation temperature of 800 °C for 3 hours (NAC-800-3). Figure 2e further shows the pore size distributions calculated based on DFT model, which shows enlarged pore diameter and broadened distribution along with increasing activation temperature and time. The pore size distribution of NAC-800-4 is similar to porous carbons prepared by KOH activation^17^,^18^,^19^. These samples contain a large fraction of micropores ranging from 0.8 to 1 nm in diameter, as well as mesopores below 4 nm. Combining with the macroporous structure, such carbons with high-level of N-doping and hierarchically porous structure are of great interest for sorption and other applications.

**Figure 3:**
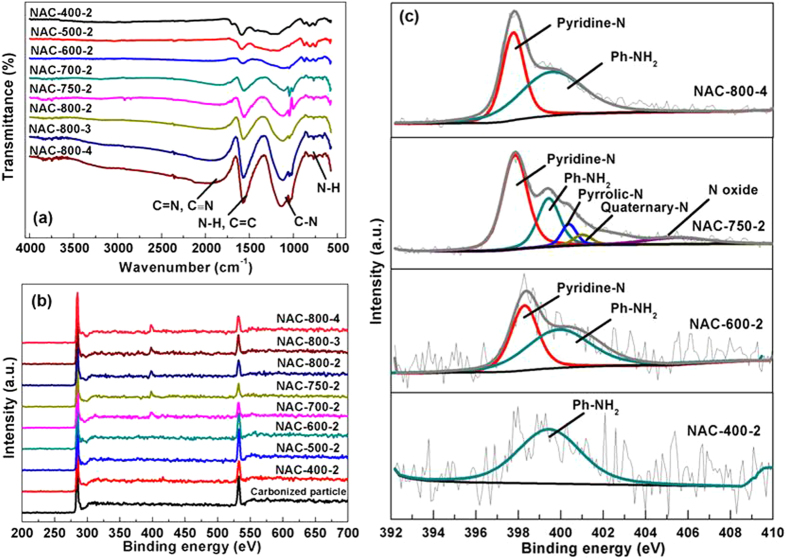
(**a**) FT-IR and (**b**) XPS spectra of all NAC samples; (**c**) N 1s XPS spectra of NAC-400-2, NAC-600-2, NAC-750-2 and NAC-800-4.

The nature of N within the carbons is investigated by Fourier transform infrared (FT-IR) spectra and X-ray photoelectron spectroscopy (XPS). FT-IR spectra (Fig. 3a) of samples shows a board band at 2000 cm^−1^, which is characteristic of the nitrile group or C=N from pyridine and quinoline^29^,^30^. The bands at ca. 1590 cm^−1^ can be assigned to the presence of N-H in-plane deformation vibration or C=C stretching vibration from aromatic rings^6^. The peaks around 1150–900 cm^−1^ are attributed to the C-N stretching vibration. The broad weak bands between 950 and 650 cm^−1^ correspond to out-of-plane N-H deformation vibration^5^,^6^. Hence, FT-IR analysis confirms the existence of numerous N-containing groups in the carbon samples.

Besides, XPS spectra of NACs (Fig. 3b) clearly shows the incorporation of N atoms into carbon framework. The bonding configurations of N atoms are further characterized by the high-resolution N1s spectra (Fig. 3c). The peaks of N1s spectra can be deconvolved and assigned to various N-containing groups, including pyridine N (398.1 eV), Ph-NH_2_ (399.4 eV), pyrrolic N (400.5 eV), quaternary N (401.3 eV) and N-oxides (402–405 eV)^5^,^6^,^7^,^8^,^31^. As shown in Fig. 3c, N-containing species within the samples change significantly with activation temperature and time. Specifically, only one peak corresponding to Ph-NH_2_ group (399.4 eV) can be seen at a low activation temperature (NAC-400-2). Two intense peaks at 398.3 and 399.7 eV can be distinguished above 600 °C, corresponding to pyridine-N and Ph-NH_2_ respectively. During the activation at 750 °C, multiple N species are formed, including pyridine-N, Ph-NH_2_, pyrrolic-N, quaternary-N and N-oxides. However, most of N-containing groups are highly volatile at high temperature. With further increased temperature and reaction time, only two N-containing groups, i.e., pyridine-N and Ph-NH_2_ remain at 800 °C (Figure S1). Noticeably, Ph-NH_2_ and pyridine-N are the most effective N-containing groups for CO_2_ capture due to the preferred interactions between CO_2_ and the electronegative N-containing groups^4^,^5^,^6^,^7^,^8^,^9^,^10^,^11^.

From the thermodynamic point of view, the direct reaction between carbon and NH_3_ (Eq. 2),
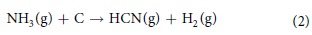


is favorable only at high temperature (e.g., △H = 188.53 kJ mol^−1^ at 1273 K)^32^,^33^. The ability to realize high-level of N-doping at low temperature can be attributed to the unique chemical composition of the corncob precursor. The main compositions of corncob are cellulose, hemicellulose and lignin^34^,^35^, possessing multiple oxygen (O)-containing groups, e.g., C–O, C=O and –OH etc., which can be identified at 900–1800 cm^−1^ in the FT-IR spectrums (Figure S2). Such groups may play important roles for N doping during NH_3_ treatment^24^,^27^. As shown in Table 1, large amounts of the O-containing groups are consumed during the initial activation process, as suggested by the obvious decrease of the O content. It is possible that NH_3_ reacts with such groups, forming amine-containing sites such as Ph-NH_2_ moieties. With increase of the activation temperature, the carbon scaffolds react with NH_3_, forming pores by transforming carbon to Hydrogen cyanide gas (HCN)^32^,^33^, during which N atoms are also doped into the aromatic rings (e.g., in the forms of C–N or C=N). As a result, the N content continually increases, accompanied by the decrease content of carbon with increasing the degree of the activation.

To confirm the role of the O-containing groups in the doping process, corncob-derived activated carbon (denoted as CAC, SSA of ~3711 m^2^g^−1^) is also prepared by the conventional KOH activation method (see preparation details in Experimental Section). Such carbon materials contain significantly lower content of the O-containing groups (~ 7% vs ~20% for the pre-carbonated corncob particles, see Table 1 and Figure S2). Consistently, treating the CAC by NH_3_ under the same condition leads to a much lower N doping (<2%) even at 800 °C, confirming the essential roles of the O-containing groups in the N-doping process. Based on the studies presented, it is reasonable to conclude that NH_3_ plays dual roles as the activating agent and N-doping source. During the activation process, such N-containing groups are dynamically generated and removed, leading to the formation of highly porous carbons with high content of Ph-NH_2_ and pyridine-N groups.

**Figure 4:**
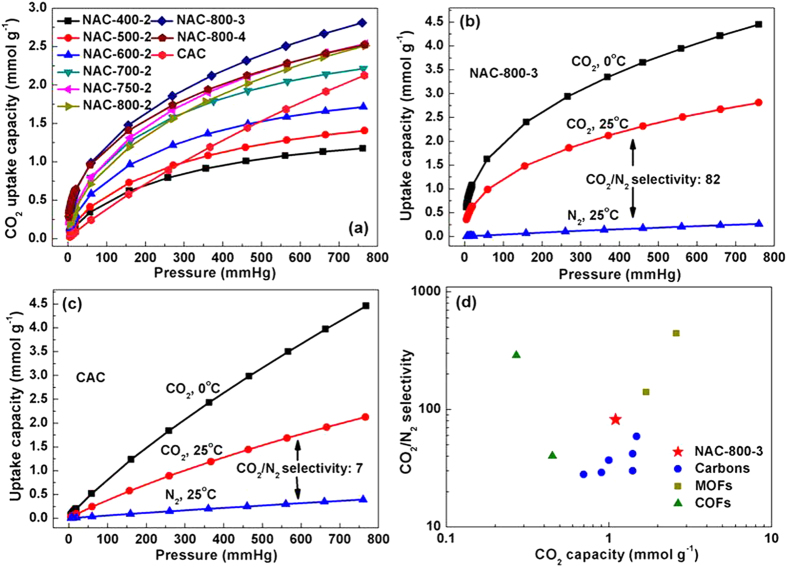
(**a**) CO_2_ adsorption isotherms of all NAC samples at 1 bar and 298 K; (**b**) Adsorption isotherms of NAC-800-3 for CO_2_ at 273 and 298 K, and N_2_ at 298 K; (**c**) Adsorption isotherms of CAC for CO_2_ at 273 and 298 K, and N_2_ at 298 K; (d) Comparison of CO_2_ adsorption capacity (298 K, 0.1 bar) and CO_2_/N_2_ selectivity of NAC-800-3 with different types of representative solid physisorbents (carbons^4^,^5^,^6^,^7^,^8^,^9^,^10^,^11^, MOFs^12^,^13^,^14^, COFs^15^,^16^).

Figure 4a shows the CO_2_ adsorption performance of as-prepared samples at 1 bar and 298 K. Remarkably, CO_2_ adsorption capacity improves with increased activation temperature and slightly increased reaction time, reaching a maximum value of 2.81 mmol g^−1^ for NAC-800-3 due to the high SSA and large amount of N-containing groups (i.e. Ph-NH_2_ and pyridine-N). For comparison, CAC is also tested for CO_2_ adsorption. Interestingly, although NAC-800-3 only holds one third of SSA of CAC, its CO_2_ adsorption capacity is about 33% higher, which reveals the crucial role of N-containing groups on CO_2_ capture.

More importantly, the N-containing groups also endow NACs with advantages on CO_2_/N_2_ selectivity. Adsorption selectivity of CO_2_ over N_2_ is calculated by the ratio of initial slopes of the CO_2_ and N_2_ adsorption isotherms (Figure S3 and Figure S4). As a result, the CO_2_/N_2_ selectivity of representative sample NAC-800-3 is about 82:1 at 298 K. To the best of our knowledge, such CO_2_/N_2_ selectivity is one of the highest values among reported nanoporous carbons (e.g., 28 for HCM-DAH-1^4^, 42 for IBN9-NCI^8^, 59 for CPC 550^9^, 124 for SU-MAC-500^36^). In addition, the ideal adsorption solution theory (IAST) selectivity (assuming gas mixture of CO_2_/N_2_ with 10% CO_2_ at 1 bar) is calculated to be 45:1 at 298 K. The value discrepancy between the initial slope method and IAST method can be attributed to the adsorption site heterogeneity^37^. As shown in Fig. 4d, although higher CO_2_/N_2_ selectivity has been achieved on other porous framework materials (e.g., 140 for SIFSIX-Cu-I at 298 K^13^, 442 for PPN6-CH_2_DETA at 298 K^14^, 288 for azo-COP at 323 K^15^), considering the low-cost, easy scalability and facile synthesis method, the NACs reported in this work are more attractive for potential industrial applications.

**Figure 5:**
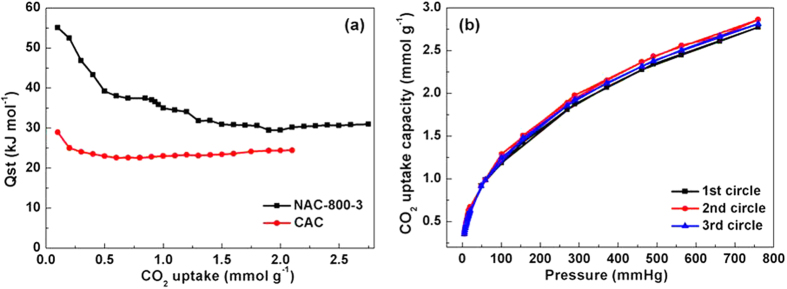
(**a**) Isosteric heat of CO_2_ adsorption for NAC-800-3 and CAC at different CO_2_ uptakes; (**b**) CO_2_ multi-circle sorption isotherms for NAC-800-3 at 298 K.

Superior CO_2_/N_2_ selectivity of NAC-800-3 is also supported by ultra-high isosteric heats of adsorption (Q_st_). Q_st_ can be calculated via the CO_2_ adsorption isotherms at 273 and 298 K (Fig. 4b,c) and applying the Clausius-Clapeyron equation (E.g. 1, see calculation details in the Experimental Section). NAC-800-3 exhibits an ultra-high Q_st_ of 55.1 kJ mol^−1^ at the initial stage and 30.5 kJ mol^−1^ at the steady stage (Fig. 5a), which is much higher than those previously reported values of porous carbons (e.g., 35.9–21.1 kJ mol^-1^ for HCM-DAH-1^4^, 44.1–27.0 kJ mol^−1^ for IBN9-NCI^8^). As a comparison, N-free CAC exhibits a lower and almost unchanged Q_st_ of 28.9–24.4 kJ mol^−1^. The ultra-high Q_st_ of NAC-800-3 at the initial stage decreases gradually at higher uptake, which indicates the presence of active sites (i.e., N-containing groups) on the surface of NAC^38^.

Overall, both surface chemical property (i.e. N-containing groups) and high SSA accompanied with microporous structure are critically important for selective adsorption of target gas molecules. Although CAC is essentially microporous and possesses ultra-high SSA, it only exhibits low selectivity of about 7:1 (Fig. 4c and Figure S4), which is obviously inferior to NAC-800-3. Hence, merely increasing SSA of carbon adsorbent is not enough for enhancing selective CO_2_ capture performance. The ultra-high Q_st_ of NAC-800-3 is due to the synergetic effects of high SSA with microporous structure and rich N-containing groups, leading to superior selective adsorption of CO_2_ over N_2_.

The reversibility of CO_2_ adsorption for NAC-800-3 at 298 K has been tested over 3 cycles (Fig. 5b). Adsorption capacities for 3 cycles are almost identical, with generally overlapped desorption and adsorption curves. Thus, CO_2_ capture in NAC is highly reversible and primarily based on physisorption. The easy regeneration of NAC under mild conditions makes it superior to aqueous amine and amine-modified solids, which require large amount of energy during the regeneration process^1^,^2^.

## Conclusion

In summary, we have developed a one-step synthesis method of N-doped microporous carbon monoliths derived from biomass using an NH_3_-assisted activation process, where NH_3_ serves as both activating agent and N source. The as-prepared N-doped active carbons (NACs) exhibit rich micropores with high surface area and high N content. Synergetic effects of its high surface area, microporous structure and high N content, especially rich N-containing groups for effective CO_2_ capture (i.e., Ph-NH_2_ and pyridine-N) lead to superior CO_2_/N_2_ selectivity up to 82, which is the highest among known nanoporous carbons. In addition, the NACs can be easily regenerated under mild conditions. Considering the outstanding CO_2_ capture performance, low production cost, simple synthesis procedure and easy scalability, NACs are promising candidates for selective capture of CO_2_ in industrial applications.

## Additional Information

**How to cite this article**: Geng, Z. *et al.* One-Step Synthesis of Microporous Carbon Monoliths Derived from Biomass with High Nitrogen Doping Content for Highly Selective CO_2_ Capture. *Sci. Rep.* **6**, 30049; doi: 10.1038/srep30049 (2016).

## Supplementary Material

Supplementary Information

## Figures and Tables

**Figure 1:**
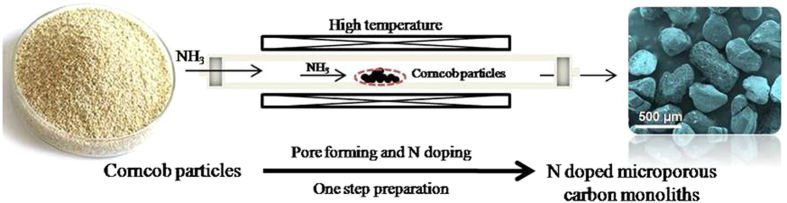
Schematic illustration about one-step synthesis of nitrogen doped microporous carbon monoliths derived from biomass corncob.

**Table 1:** Textural and chemical characteristic of various samples.

**Samples**	**Pore properties**			**Elemental analysis**			**Yield(wt%)**
	**SSA**^a^**(m**^**2**^**g**^**−1**^)	**V**_**t**_^b^ **(cm**^**3**^**g**^**−1**^)	**C(wt%)**	**N(wt%)**	**O(wt%)**	**H(wt%)**	
Corncob	–	–	44.87	0.38	48.43	6.32	–
Carbonized particle	–	–	75.15	<0.3	20.2	4.35	–
CAC	3711	2.07	91.63	<0.3	7.39	0.38	32.34
NAC-400-2	–	–	78.80	1.47	16.59	3.14	86.8
NAC-500-2	–	–	82.44	1.82	12.90	2.84	77.68
NAC-600-2	–	–	83.70	3.88	10.37	2.05	78.04
NAC-700-2	494	0.16	78.21	9.82	9.16	1.41	77.21
NAC-750-2	784	0.27	78.11	10.59	9.84	1.46	67.63
NAC-800-2	1086	0.44	75.43	10.82	12.29	1.46	45.24
NAC-800-3	1154	0.57	69.10	11.52	17.88	1.50	27.62
NAC-800-4	1027	0.53	65.67	12.30	20.52	1.91	17.13

^a^SSA, specific surface area calculated by BET equation at P/P_o_ = 0.02–0.25. Correlation coefficient of BET curves for all samples is higher than 0.9999.

^b^V_t_, total pore volume estimated from the adsorption amount of N_2_ at P/P_o_ = 0.98.
