# Supplementary material for: One-Step Synthesis of Microporous Carbon Monoliths Derived from Biomass with High Nitrogen Doping Content for Highly Selective CO2 Capture
Source: Sci Rep. 2016 Aug 4;6:30049. doi: 10.1038/srep30049 (PMC4973261; doi:10.1038/srep30049)
Supplement: Supplementary Information [file srep30049-s1.doc]

Supporting Information

**One-Step Synthesis of Microporous Carbon Monoliths Derived from Biomass with High Nitrogen Doping Content for Highly Selective CO_2_ Capture**

*Zhen Geng^＋^, Qiangfeng Xiao^＋^, Hong Lv, Bing Li, Haobin Wu, Yunfeng Lu and Cunman Zhang**


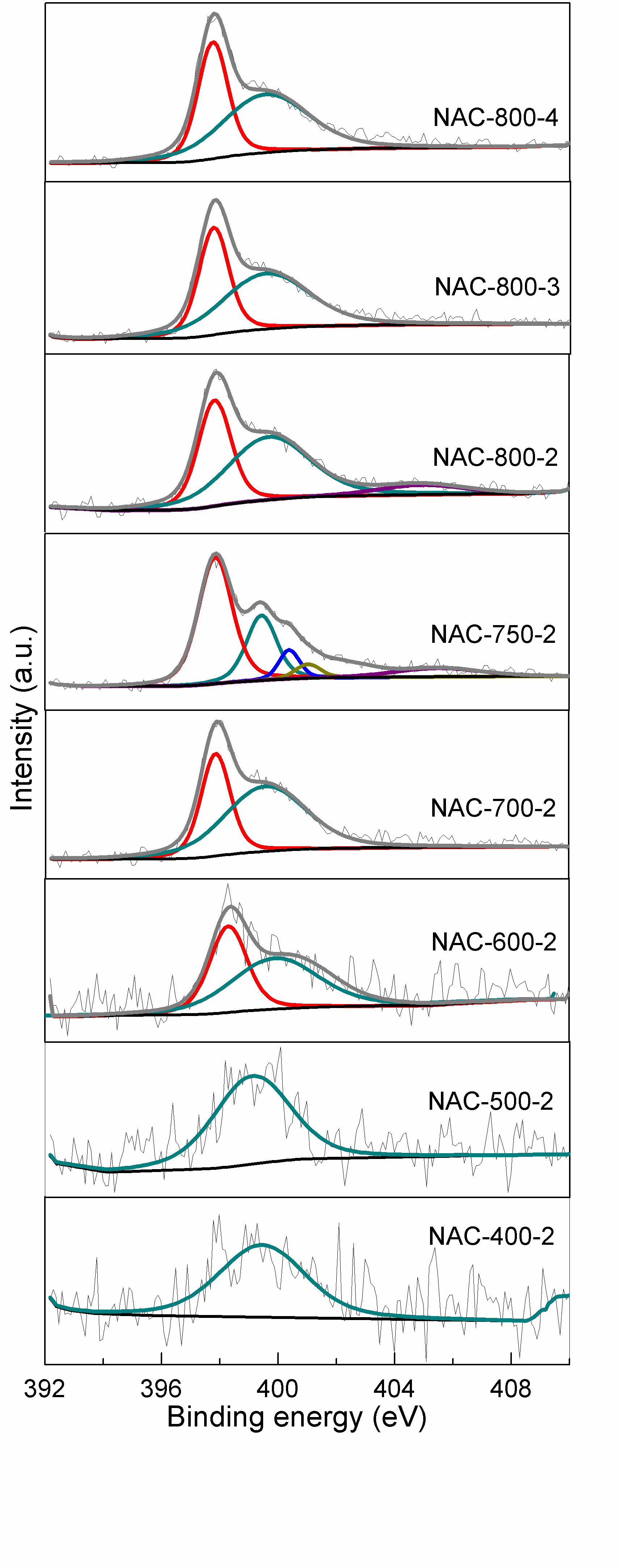


**Figure S1.** N 1s XPS spectra of all as-obtained samples.


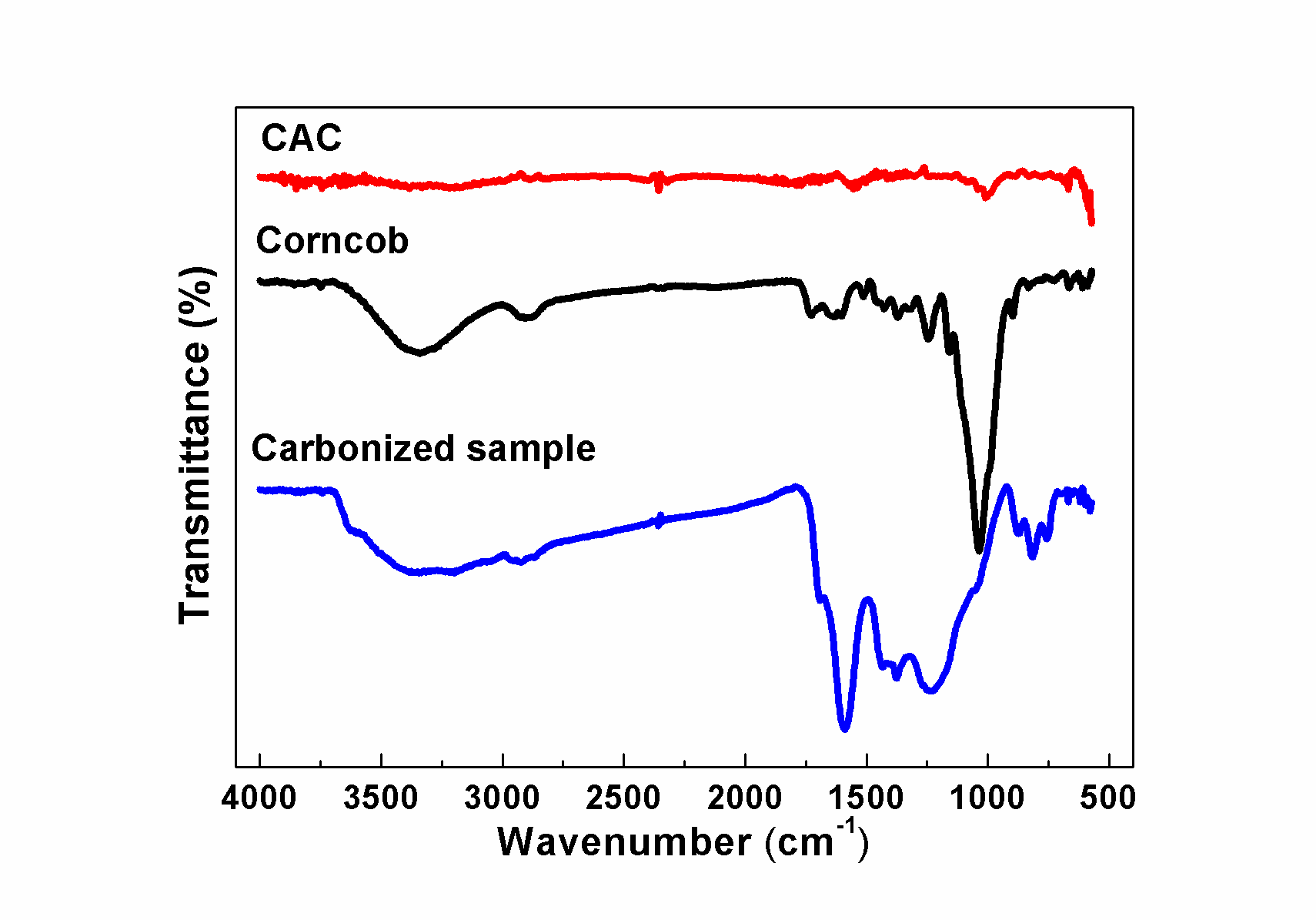


**Figure S2.** FT-IR spectra of corncob powders, carbonized sample and CAC prepared by KOH activation.


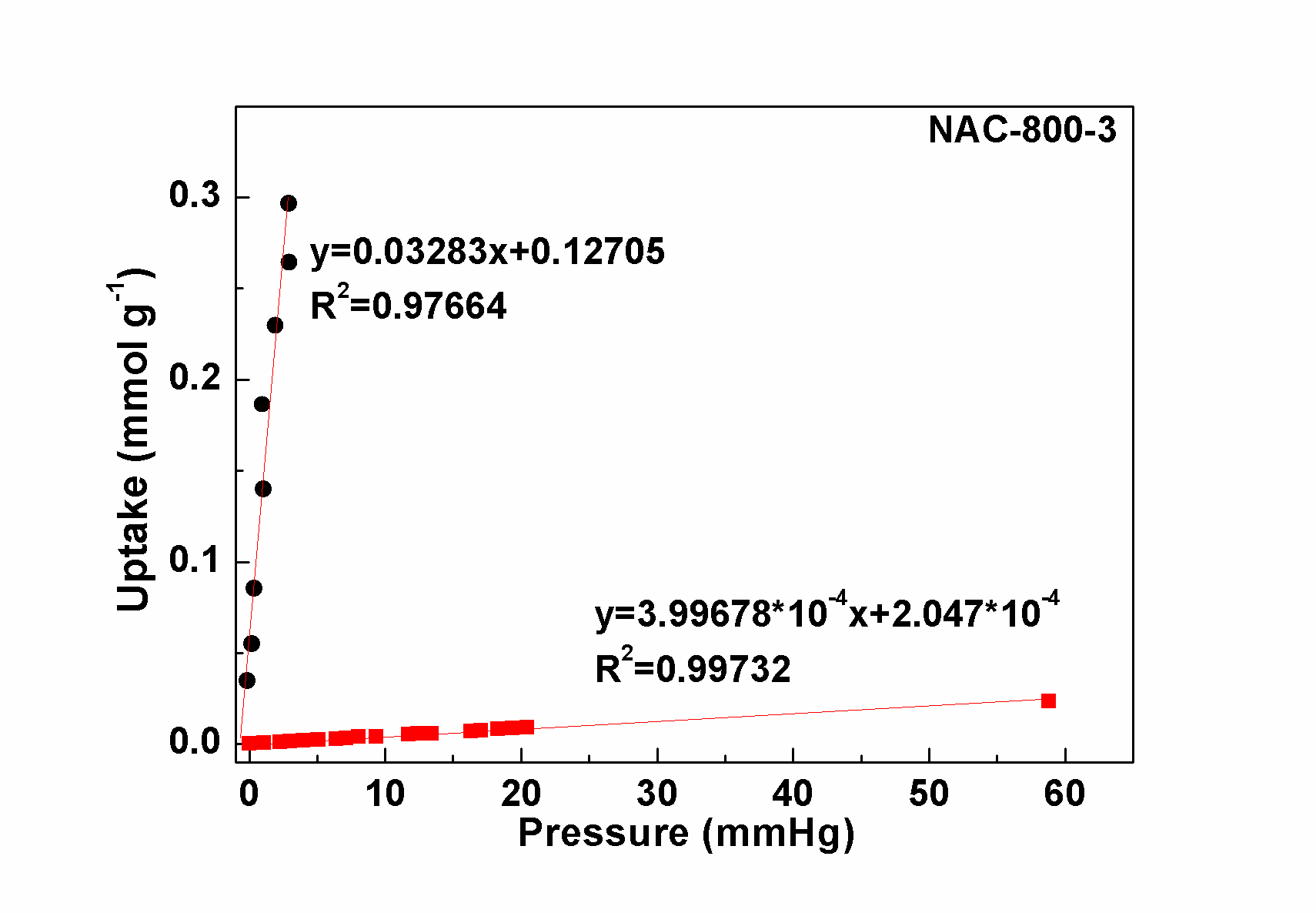


**Figure S3.** Initial slope calculation for CO_2_ and N_2_ uptake isotherms for NAC-800-3 at 298 K (CO_2_: black circles; N_2_: red squares).


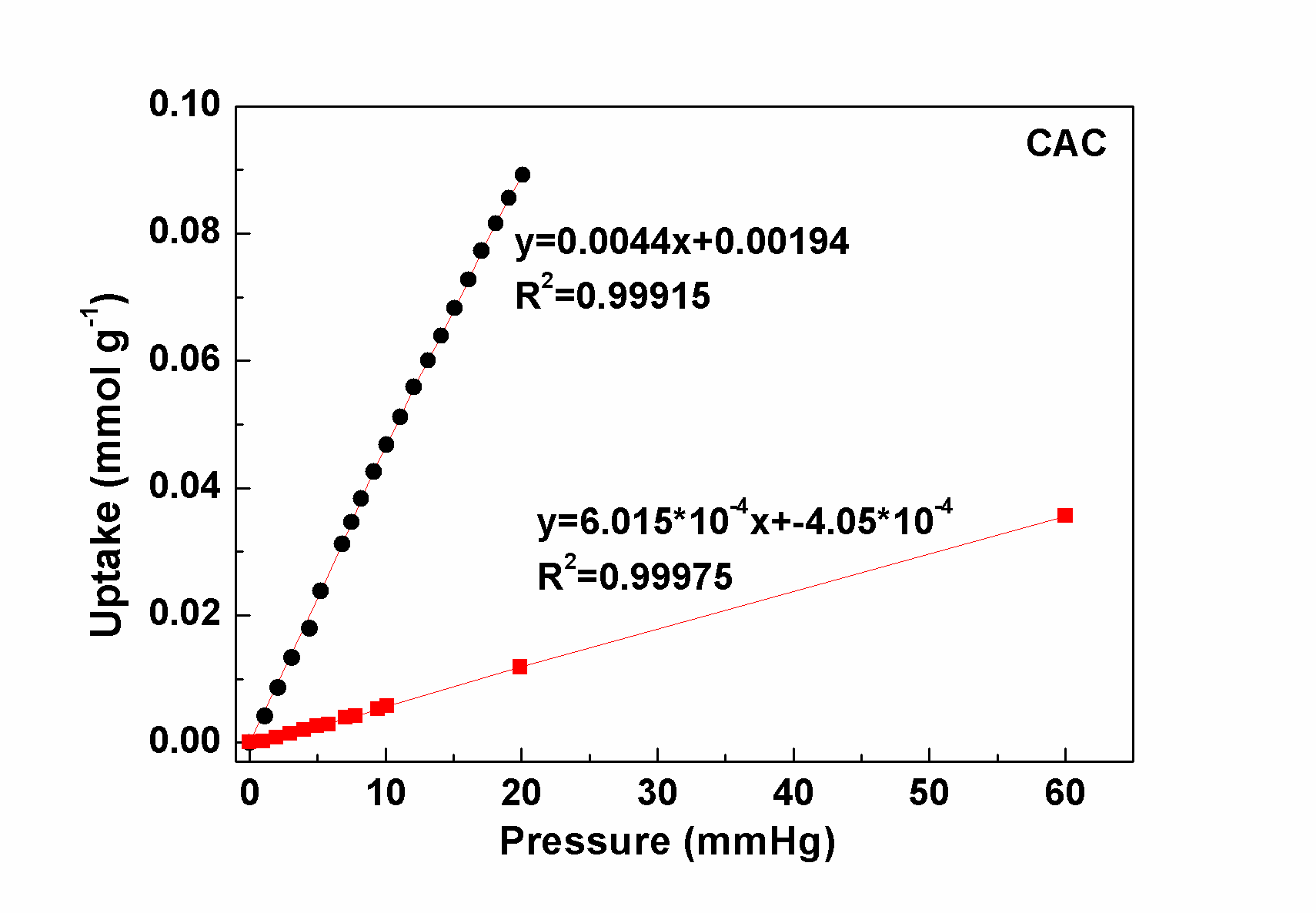


**Figure S4.** Initial slope calculation for CO_2_ and N_2_ uptake isotherms for CAC at 298 K (CO_2_: black circles; N_2_: red squares).
